# Effectiveness of Resiliency and Recovery Program on Compassion Fatigue among Nursing Officers working in selected Hospitals in India

**DOI:** 10.17533/udea.iee.v41n3e06

**Published:** 2023-10-21

**Authors:** Betsy Sara Zacharias, Sheela Upendra

**Affiliations:** 2 Deputy Director and Professor. Email: sheelaupendra@scon.edu.in sheelaupendra@scon.edu.in; 3 Symbiosis College of Nursing. Symbiosis International (Deemed University), Pune. India Symbiosis International University Symbiosis College of Nursing Symbiosis International (Deemed University) Pune India

**Keywords:** burn out, professional, compassion fatigue, nurses, agotamiento professional, desgaste por empatía, enfermeras y enfermeros, esgotamento profissional, fadiga por compaixão, enfermeiras e enfermeiros

## Abstract

**Objective.:**

The study objective was to evaluate the effectiveness of Resiliency and Recovery Program on Compassion Fatigue level of Nursing Officer from selected hospitals of Pune City (India).

**Methods.:**

The study used a quasi-experimental approach involving single group pre-test and post-test design. 100 nursing officers, working in selected hospitals of Pune city, who were willing to participate were selected using non probability convenience sampling. The data was collected using *The Professional Quality of Life Scale: Compassion Satisfaction and Fatigue* (ProQoL) Version 5 of Stamm. The study included pre-test, resiliency and recovery program and post-test. Resiliency and Recovery Program is an intervention aiming to develop five resiliency skills or antibodies including (a) self-regulation, (b) perceptual maturation, (c) intentionality, (d) self-care and (e) connection and support.

**Results.:**

Statistically significant difference was revealed between the pre-test and post-test score means: Compassion Satisfaction (pre-test = 28.50 to post-test = 31.0; t-18.6671, *p*<0.001), Burn-out (pre-test = 35.2 to post-test = 31.7; t-15.00, *p*<0.001), and Secondary Traumatic Stress (pre-test = 37.4 to post-test = 33.07; t-14.8996, *p*<0.001).

**Conclusion.:**

Resiliency and Recovery Program had a significant impact on Compassion Fatigue, leading to an increase in Compassion Satisfaction, and a reduction in Burnout and Secondary Traumatic Stress. Inculcating Resiliency skills in nursing officers can help them in reducing compassion fatigue and thus aids in health promotion.

## Resumen

**Objetivo.:**

El objetivo del estudio era evaluar la eficacia del Programa de Resiliencia y Recuperación en el nivel de Fatiga por Compasión de los profesionales de enfermería de los hospitales seleccionados de la ciudad de Pune (India).

**Métodos.:**

El estudio cuasi-experimental con evaluación pre y post-intervención en un solo grupo. Se seleccionaron 100 profesionales de enfermería que trabajaban en hospitales seleccionados de la ciudad de Pune mediante un muestreo no probabilístico por conveniencia. Los datos se recogieron utilizando la *Escala de calidad de vida profesional*: *Compassion Satisfaction and Fatigue (*(ProQOL) Version 5 de Stamm. El estudio incluyó una prueba previa, un programa de resiliencia y recuperación y una prueba posterior. El Programa de Resiliencia y Recuperación es una intervención cuyo objetivo es desarrollar cinco habilidades o anticuerpos de resiliencia que incluyen (a) autorregulación, (b) maduración perceptiva, (c) intencionalidad, (d) autocuidado y (e) conexión y apoyo.

**Resultados.:**

Se observaron diferencias estadísticamente significativas entre las puntuaciones promedio obtenidas antes y después de la intervención en la satisfacción con la compasión (antes = 28.5 a después = 31; t-18.6671, *p*<0.0001), el agotamiento (antes = 35.2 a después = a 31.7; t-15,00, *p*<0.001) y el estrés traumático secundario (antes = 37.4 a después 33.1; t-14.8996, *p*<0.001).

**Conclusiones.:**

El Programa de Resiliencia y Recuperación tuvo un impacto significativo en la Fatiga por Compasión, lo que condujo a un aumento de la Satisfacción por Compasión y a una reducción del Burnout y del Estrés Traumático Secundario. Inculcar habilidades de resiliencia a los profesionales de enfermería puede ayudarles a reducir la fatiga por compasión y, por tanto, a promover la salud.

## Resumo

**Objetivo.:**

O objetivo do estudo foi avaliar a eficácia do Programa de Resiliência e Recuperação no nível de Fadiga por Compaixão em profissionais de enfermagem em hospitais selecionados na cidade de Pune (Índia).

**Métodos.:**

Foi realizado um estudo quase experimental com avaliação pré e pós-intervenção em grupo único. Foram selecionados 100 profissionais de enfermagem que trabalham em hospitais da cidade de Pune por meio de amostragem não probabilística de conveniência. Os dados foram coletados por meio da versão 5 da Escala de Qualidade de Vida Profissional: Compaixão, Satisfação e Fadiga (ProQoL) de Stamm. O estudo incluiu um pré-teste, um programa de resiliência e recuperação e um pós-teste. O Programa de Resiliência e Recuperação consistiu em uma intervenção cujo objetivo é desenvolver cinco habilidades de resiliência ou anticorpos que incluem (a) autorregulação, (b) maturação perceptual, (c) intencionalidade, (d) autocuidado e (e) conexão e suporte.

**Resultados.:**

Foram observadas diferenças estatisticamente significativas entre as pontuações médias obtidas antes e depois da intervenção em satisfação por compaixão (antes = 28.5 a depois = 31; t-18.6671, *p*<0.0001), burnout (antes = 35.2 a depois = a 31.7; t-15.00), *p*<0.001) e estresse traumático secundário (antes = 37,4 a depois 33.1; t-14.8996, *p*<0.001).

**Conclusões.:**

O Programa de Resiliência e Recuperação teve um impacto significativo na Fadiga por Compaixão, levando a um aumento na Satisfação por Compaixão e a uma redução no Burnout e no Estresse Traumático Secundário. Incutir competências de resiliência nos enfermeiros pode ajudá-los a reduzir a fadiga da compaixão e, portanto, promover a saúde.

## Introduction

A nurse is constantly exposed to the trauma and suffering of others as a part of professional life. And it is almost impossible that their personal life is not impacted by it. According to Lombardo and Eyre,^1^ an empathetic nurse becomes victim of the constant stress of meeting the requirements of patients and their family members, leading to compassion fatigue, which affects the nurse in terms of physical and emotional health. Compassion fatigue reduces job satisfaction as well as leads to decreased productivity and increased turnover”. 

Figley^2^ coined the term *Compassion Fatigue* and according to him compassion fatigue occurs because of continuous exposure to chronic stress in caring for patients who go through pain, grief, catastrophe, and misery. Figley defined Compassion Fatigue as “a state of tension and preoccupation with the individual or cumulative trauma of clients as manifested in the following ways: Re-experiencing the painful events, avoidance of reminders of the traumatic event, persistent arousal, Combined with the effects of cumulative stress (burnout)”. Joinson^3^ describes Compassion Fatigue as being “emotionally devastating.” Due to compassion fatigue nurses experience problems like anger, detachment, depression, dreading going to work, feeling irritable, fatigue, a lack joyful affect, and physical complaints such as frequent headache and stomach ache. This can ultimately lead to decreased productivity at work and job satisfaction and thus patients receive a lower standard of nursing care. Compassion fatigue is a severe emotional pain which is related to the physical, emotional, and spiritual fatigue which is going beyond the individual and leading to a subtle turn down in the vigour to care for one and others. 

Nursing officers working with suffering individuals often share the emotional burden of people they care for. This indirect exposure to pain, suffering, and trauma involves an inherent risk of significant cognitive, behavioural, and emotional changes in the Nursing officer and makes them vulnerable to developing Compassion Fatigue Few risk factors in developing compassion fatigue are as follows: high levels of stress, history of trauma, negative coping skills, low levels of social support, and bottling up of emotions or avoiding expression of emotions.^4^ Compassion Fatigue is a burning issue and requires careful attention and intervention. Lee and Hen^5^ studied prevalence of burn out among 1896 Taiwanese nurses, and they identified that 79% of the nurses had burn-out. Rajeswari and Sreelekha^6^ studied the level of stress among 200 nurses working in a tertiary care hospital in India, and identified that 59.5% of them had high level of stress_._ Duffy *et al.*^7^ assessed secondary traumatic stress among emergency nurses and concluded that 64% met the criteria for secondary traumatic stress. Rajeswari^8^ examined the level of burnout among 200 nurses employed in a tertiary care hospital and found that 54% of them had severe burnout.

Resiliency and Recovery Program is an intervention aiming to develop five resiliency skills or antibodies including (a) self-regulation, (b) perceptual maturation, (c) intentionality, (d) self-care and (e) connection and support. Self-regulation is the ability to intentionally control the activity of one’s Autonomic Nervous System while involved in the daily activities and thus lessen the energy spend. It includes consistently moving away from the constant activation and dominance of Sympathetic Nervous System (SNS) towards the comfortable relaxation of Parasympathetic Nervous System (PNS). Intentionality is the opposite of reactivity. It includes deliberateness and integrity. Perceptual maturation is more cognitive skill than behavioural skill. It involves maturation of our perception of workplace to making them less stressful. Feeling heard, supported, cared about and rightly understood by peers is the vital element of connection and support to maintain resiliency and to defeat Compassion Fatigue. The researcher is interested to study the effectiveness of Resiliency and Recovery Program on compassion fatigue among nursing officers because Compassion Fatigue is often overlooked and neglected. Taking care of the mental health of the nurse who works at the bed side is the need of the hour. The objective of this study was to evaluate the effectiveness of Resiliency and Recovery Program on Compassion Fatigue level of Nursing Officers from selected hospitals of Pune City (India).

## Methods

The study used quantitative quasi experimental approach involving single group pre-test and post-test design samples included 100 nursing officers from selected hospitals of Pune city, India. Permission for conducting the research was taken from concerned authorities of the Hospitals from where sample was collected. Non probability convenience sampling was used to select the samples. This sampling method was adopted since the researcher had limitations with respect to time and fund. The participants signed a written informed consent, after sufficient explanation regarding the purpose and process of the study.

The tool for data collection had two sections, Demographic data, and The Professional Quality of Life Scale: Compassion Satisfaction and Fatigue Version 5 (ProQoL),^9^ which has three subscales namely Compassion Satisfaction (CS), Burnout (BO), and Secondary Traumatic Stress (STS). ProQoL is a standardized tool and permission was obtained to use it. It has 30 questions out of which 10 questions are intended to measure compassion satisfaction, 10 questions for burn-out and 10 questions for secondary traumatic stress. In each subscale the sum of actual scores is obtained by adding up the score in the 10 items and then the score is equalized to the raw score and it is interpreted as low, average, and high. The minimum score of each subscale is 10 and the maximum score is 50. Professional quality of life is interpreted as high when the score in compassion satisfaction subscale is high, score in both burnout as well as secondary traumatic stress are low, average when the scores are average in in all three sub scales and low when the score in compassion satisfaction subscale is low, score in both burnout as well as secondary traumatic stress are high.

Psychometric properties of the scale. The alpha reliabilities for the scales were: Compassion Satisfaction alpha = 0.87, Burnout alpha = 0.72 and Compassion Fatigue alpha = 0.80. PROQoL depicted good construct, convergent and discriminant validity.

Research study included pre-test, Resiliency and Recovery Program and post-test. The participants were nursing officers from three hospitals in Pune, India selected based on the eligibility criteria of willingness and availability for study. The intervention was Resiliency and Recovery Program which is a training to develop five resiliency skills or antibodies including (a) self-regulation, (b) perceptual maturation, (c) intentionality, (d) self-care and (e) connection and support. Outcome was assessed by measuring the increase in mean compassion satisfaction score and decrease mean scores of burnout and secondary traumatic stress. The data analysis was done with Mean, standard deviation, paired t test and chi square test. 

Ethical Consideration: Institutional Research Committee clearance obtained, Permission for conducting the research was taken from concerned authorities of the Hospitals from where sample was collected. The participants signed a written informed consent, after sufficient explanation regarding the purpose and process of the study. Sample Confidentiality was maintained. 

## Results

100 nurses were selected for the research from selected hospitals of Pune City. 

**Figure 1:**
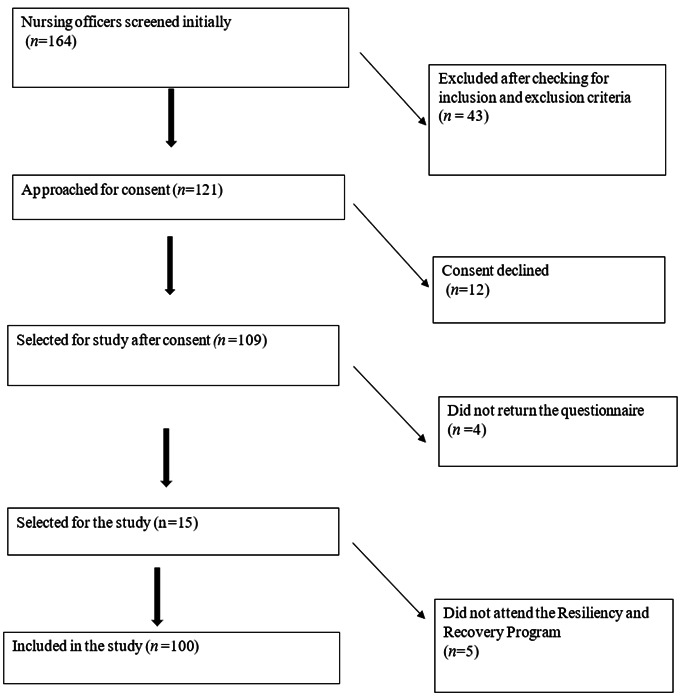
Participant flow diagram

Recruitment: 100 nursing officers from three hospitals attended the sessions in four groups of 25 each. On day one pre-test was conducted using the tool. The data was examined using frequency, percentage, mean and standard deviation to understand the level of compassion satisfaction, burn out and secondary traumatic stress. On day 2 the session on Resiliency and Recovery Program was conducted and post test was conducted after completion of five weeks. The same schedule was followed for all four groups. (Figure 1)

Analysis of Demographic variables. 12 demographic variables were considered in the study. Table 1 describes the frequency of population based on their personal characteristics. Majority of the nurses belonged to the age group 21 -25 years. 63% were females. 69% of them had completed General Nursing and Midwifery course. 58% of them had 1-5 years of experience. Majority of them were working in general wards.

**Table 1:** Distribution of population based demographic data (*n*=100)

Demographic variable	Frequency and %
Age	
21 - 25 years	51
26 - 30 years	33
31 - 35 years	11
36 - 40 years	5
Gender	
Male	37
Female	63
Marital Status	
Married	35
Unmarried	49
Divorced	3
Widowed	3
Professional Qualification	
G.N.M	69
B.Sc. Nursing	20
PB.B.Sc. Nursing	11
Years of Experience	
1-5 years	58
6-10 years	34
11 - 15 years	8
Area of Work	
Casualty	26
ICU/HDU	19
General Ward	55
Area of living	
Urban	68
Rural	32
Type of family	
Joint	22
Nuclear	46
Extended	32
Selection of profession with interest	
Yes	78
No	22
Coping Strategies Adopted	
Listening to music	24
Reading	11
Social media	54
Religious activity	7
Sleeping	4
History of any physical illness *	
Yes	23
No	77
Any recent loss	
Yes	18
No	82

* Such as Asthma, Diabetes Mellitus, Hypertension, Anaemia etc

Section 2. Analysis of Compassion Fatigue among Nursing Officers. In pre-test, in all subscales of ProQOL, moderate levels were more frequent. It should be noted that a significant proportion of participants had high levels of the subscales of compassion fatigue, as follows: 23% of them had low compassion satisfaction, 19% had high burnout and 22% had high level of Secondary Traumatic Stress. Table 2 represents the distribution of population according to score in ProQOL. 

**Table 2:** Distribution of population according to score in ProQOL (*n*=100)

Score Level	Compassion Satisfaction	Burnout	Secondary Traumatic Stress
Low (<=22); %	23%	12%	8%
Moderate (23-41); %	71%	69%	70%
High (≥42) ; %	6%	19%	22%
Total; mean±SD)	28.50±7.30	35.20±6.90	37.40±6.46

Section 3. Analysis of effectiveness of Resiliency and Recovery Program on compassion fatigue level among Nursing Officers. Analysis of the data indicated changes in the compassion fatigue level of nursing officers after the participation in Resiliency and Recovery Program. Researcher applied paired t-test for the effectiveness of Resiliency and Recovery Program on Compassion satisfaction, burn out and secondary traumatic stress among Nursing Officers. Average compassion satisfaction score among nursing officers in pre-test was 28.50 which increased to 31.01 in post-test. t-value for this test was 18.6671 with 99 degrees of freedom. Corresponding p-value was small (less than 0.05), the null hypothesis is rejected. Average burnout score among nursing officers in pre-test was 35.20 which decreased to 31.70 in post-test. t-value for this test was 15.00 with 99 degrees of freedom. Corresponding p-value was small (less than 0.05), the null hypothesis is rejected. Average Secondary Traumatic Stress score among nursing officers in pre-test was 37.40 which reduced to 33.07 in post-test. t-value for this test was 14.8996 with 99 degrees of freedom. Corresponding p-value was small (less than 0.05), the null hypothesis is rejected. It is evident that the burnout and secondary traumatic stress among nursing officers reduced significantly after Resiliency and Recovery Program as well as compassion satisfaction increased. Resiliency and Recovery Program is significantly effective in improving the compassion satisfaction and reducing burn out and secondary traumatic stress among nursing officers. Table 3 shows the change in score ProQOL after Resiliency and Recovery Program and Table 4 reveals the result of paired t test for assessing the effectiveness of Resiliency and Recovery Program on compassion fatigue level among Nursing Officers. The result shows that in all three subscales there was a statistically significant difference between pre and post measurements. The score increased in subscale Compassion Satisfaction while it decreased in subscales Burnout and Secondary Traumatic Stress.

**Table 3:** Distribution of population based on pre-test and post-test score in ProQOL (*n*=100)

Level	Compassion Satisfaction		Burnout		Secondary Traumatic Stress	
	Pre-test (%)	Post-test (%)	Pre-test (%)	Post-test (%)	Pre-test (%)	Post-test (%)
Low	23	10	12	17	8	10
Moderate	71	78	69	76	70	83
High	6	12	19	7	22	7

**Table 4:** Result of paired t-test (*n*=100)

Sub scale	Group	Mean	SD	SEM	t- value	df	***p*-value**
Compassion Satisfaction	Pre-test	28.50	7.30	0.73	18.6671	99	0.0001
Post-test	31.01	7.23	0.72
Burnout	Pre-test	35.20	6.90	0.69	15.00	99	0.0001
	Post-test	31.70	6.79	0.68			
Secondary Traumatic Stress	Pre-test	37.40	6.46	0.65	14.8996	99	0.0001
Post-test	33.07	6.33	0.63

Section 4. Analysis of association between selected Demographic Variables and Compassion fatigue level among Nursing Officers. Marital Status, Selection of profession with interest, History of physical illness and Years of experience were found to have significant association with Compassion Satisfaction among nursing officers. Selection of profession with interest, History of physical illness were found to have significant association with Burn-out among nursing officers. Professional Qualification, Selection of profession with interest, History of physical illness and Years of experience were found to have significant association with Secondary traumatic stress among nursing officers. The result of chi square test for association is depicted in Table 5. A *p*-value less than 0.05 indicates statistically significant association between the variables.

**Table 5:** Result of chi square test

Demographic Variable	Compassion Satisfaction			Burnout			Secondary Traumatic Stress		
	(2 value	DF	*p*-value	(2 value	DF	*p*-value	(2 value	DF	*p*-value
Marital Status	16.78	3	0.01	7.94	3	0.241	8.90	3	0.178
Professional Qualification	4.89	2	0.29	6.30	2	0.177	10.03	2	0.039
Selection of profession with interest	32.72	1	<0.0001	15.85	1	<0.001	24.59	1	<0.0001
Years of experience	10.80	2	0.028	8.30	2	0.089	9.74	2	0.045
History of any physical illness	8.06	1	0.017	35.55	1	<0.0001	10.21	1	<0.01

## Discussion

In the current study, the improvement in the score of compassion satisfaction, and reduction in the scores of burn-out and secondary traumatic stress among nursing officers is statistically significant. The mean score of compassion satisfaction, burnout, and secondary traumatic stress among nursing officers in the pre-test was 28.5, 35.2, 37.4 and in post-test 31.01, 31.70, 33.07 respectively. The study concludes that resiliency and recovery programme is effective in reducing compassion fatigue. The study findings can be applied in planning various staff development programs for nurses. 

Current study results are in line with the study conducted by Potter et. *al,*^10^ to find the effectiveness of compassion fatigue resiliency programme on 13 oncology nurses working in cancer centre, US. The study concludes that compassion fatigue resiliency programme is effective in managing compassion fatigue. The present study is also supported by research conducted by Daxesh,^11^ to examine the effect of guided imagery on burnout among 60 nurses Vadodara. They concluded that guided imagery is effective in reducing burnout among nurses. The present study is also supported by the result from a study conducted by Muliira and Ssendikadiwa^12^ to assess professional quality of life of Ugandan midwives, which showed that midwives had average level of burnout 88%, compassion satisfaction 68%, and compassion fatigue (STS) levels 68%. And the mean score on the ProQOL showed compassion satisfaction was 19, burnout was 36.9 and secondary traumatic stress was 22.9. 

According to a study done by Kumari and Bist,^13^ to identify the prevalence of burnout, compassion fatigue, and compassion satisfaction among staff nurses in selected hospitals of Gautam Buddh Nagar, majority of 43 (86%) midwife nurses were having average level of compassion fatigue (STS), average level of burnout was found in 33 (66%) of midwife nurses and 16 (32%) midwife nurses had high level of compassion satisfaction. Smart *et al.* ^(^^14^ concluded that Caregivers working in a noncritical area scored less whereas the caregiver working in critical area scored high in the subscale of burnout in the professional quality of life. 

Limitations. The study did not have randomization and control group.

Conclusion. The present study concludes that participant’s nurses were vulnerable to compassion fatigue and Resiliency and Recovery Programme was effective in reducing the level of burn out and secondary traumatic stress. Hence it must be further studied and intervention must be undertaken to prevent development of compassion fatigue.

Recommendation. In-service programs focusing on resiliency skill training must be conducted for the nursing officers to reduce compassion fatigue and Compassion Fatigue and its prevention and management must be included in the syllabus for undergraduate nursing students.

## Data Accessibility:

The corresponding author can supply the dataset on reasonable request.
